# AI/Machine Learning and Sol-Gel Derived Hybrid Materials: A Winning Coupling

**DOI:** 10.3390/molecules30143043

**Published:** 2025-07-20

**Authors:** Aurelio Bifulco, Giulio Malucelli

**Affiliations:** 1 Department of Chemical, Materials and Industrial Production Engineering (DICMaPI), University of Naples Federico II, Piazzale Tecchio 80, 80125 Napoli, Italy; aurelio.bifulco@unina.it; 2 Department of Applied Science and Technology, Politecnico di Torino, Viale Teresa Michel 5, 15121 Alessandria, Italy; 3 Consorzio Interuniversitario Nazionale per la Scienza e Tecnologia dei Materiali (INSTM) Unit, Via G. Giusti 9, 50121 Florence, Italy

**Keywords:** sol-gel method, hybrid materials, artificial intelligence, machine learning, decision trees, neural networks

## Abstract

Experimental research in the field of science and technology of polymeric materials and their hybrid organic-inorganic systems has been and will continue to be based on the execution of tests to establish robust structure-morphology-property-processing correlations. Although absolutely necessary, these tests are often time-consuming and require specific efforts; sometimes, they must be repeated to achieve a certain reproducibility and reliability. In this context, the introduction of methods like the Design of Experiments (DoEs) has made it possible to drastically reduce the number of experimental tests required for a complete characterization of a material system. However, this does not seem enough. Indeed, further improvements are being observed thanks to the introduction of a very recent approach based on the use of artificial intelligence (AI) through the exploitation of a “machine learning (ML)” strategy: this way, it is possible to “teach” AI how to use literature data already available (and even incomplete) for material systems similar to the one being explored to predict key parameters of this latter, minimizing the error while maximizing the reliability. This work aims to provide an overview of the current, new (and up-to-date) use of AI/ML strategies in the field of sol-gel-derived hybrid materials.

## 1. Introduction

The scientific literature is rich with several interesting examples regarding the synthesis and characterization of sol-gel-derived hybrid organic-inorganic materials: indeed, these systems can be quite easily obtained by exploiting the occurrence of hydrolysis and condensation reactions of suitable precursors, possibly in the presence of coupling agents [1,2,3,4]. Additionally, the wide availability of the latter, together with the possibility of designing formulations with diverse types of monomers (epoxy, (meth)acrylic, vinyl ester, …), allows for obtaining hybrid materials that, at least potentially, are suitable for different applications in advanced sectors [5,6,7,8,9,10,11,12,13].

Despite their robustness and feasibility, the optimization of sol-gel-derived hybrid organic-inorganic systems requires the design, development, and implementation of testing activities aimed at identifying the best performing recipes and the best experimental parameters as well. Indeed, any changes in the starting formulations (chemical structure and reactivity of selected monomers and precursors, monomers to precursors ratio, type and loading of coupling agents, among others) and in the experimental conditions (pH, temperature, reaction time) may significantly affect the overall sol-gel process and, subsequently, the properties of the final hybrid materials [14,15,16,17]. However, this optimization is often time-consuming and requires specific efforts, also considering that experimental testing may necessitate some replicas to achieve acceptable reproducibility and reliability.

Notwithstanding that the experimental activities carried out on any material systems are remarkably necessary, an important reduction in the number of the experimental tests needed for a complete characterization has been achieved by proposing such methods as the Design of Experiments (DoEs) [18]. Briefly, DoE represents a branch of applied statistics, which encompasses planning, running, and analyzing controlled tests to evaluate factors affecting the value of one or more parameters. It is, therefore, a powerful tool for collecting and analyzing data in a variety of experimental conditions; besides, it accounts for a reduction in the experimental testing efforts, hence simplifying the experimental activities to be performed.

This methodology has also been successfully applied in the case of sol-gel-derived hybrid materials.

In particular, Mahmoodian and co-workers [19] synthesized organic–inorganic hybrid systems based on a modified Bisphenol A diglycidyl methacrylate (bearing two ethoxy-silane reactive groups), tetraethylorthosilicate (TEOS), and triethylene glycol dimethacrylate, suitable for orthodontic purposes (i.e., dental resins). The DoE approach based on the Taguchi orthogonal method was exploited to find the best experimental conditions for achieving the highest gel content without complete gelation of the resin mixture.

Najafabadi et al. [20] exploited the DoE methodology based on Taguchi orthogonal design to investigate and optimize compositional and process parameters of hybrid organic-inorganic coatings, sol-gel-derived from TEOS and 3-glycidoxypropyltrimethoxysilane, and deposited onto an Aluminum 5083 alloy. The multifactor analysis of variance (ANOVA) analysis method was employed [21], and pull-off tests assessing the adhesion strength of coatings to the metal substrate were utilized as a response.

In further research [22], the same group exploited DoE based on Taguchi orthogonal design and the ANOVA analysis method to optimize the same sol-gel-derived hybrid organic-inorganic coatings deposited onto an Aluminum 5083 alloy, using the corrosion current density as a response.

Yahyaei et al. [23] investigated the impact of diverse parameters (namely, hydrolysis ratio, molar ratio of precursors, weight percentage of inorganic to organic part, post-curing temperature, and time) on sol-gel derived hybrid UV-curable coatings synthesized from UV-curable urethane acrylates (organic phase) and TEOS and 3-methacryloxypropyl trimethoxy silane (inorganic phase). For this purpose, again, a DoE based on Taguchi orthogonal design was used to identify the best experimental conditions for obtaining hybrid systems with high abrasion resistance and, at the same time, high transparency.

**Figure 1:**
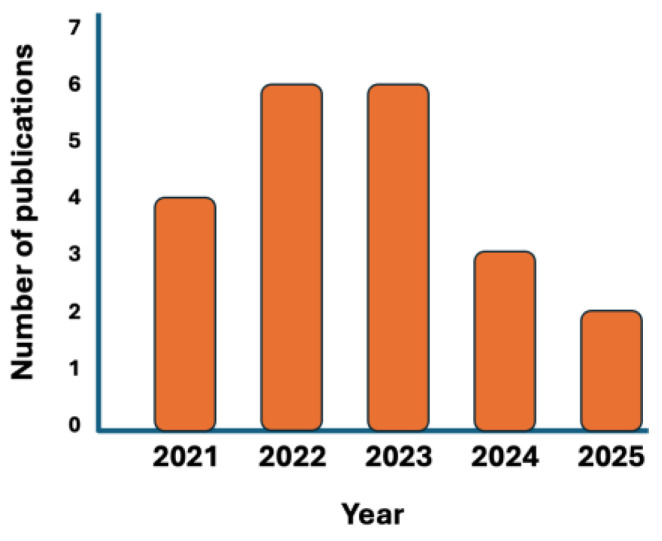
Number of publications per year dealing with “sol-gel” AND “machine learning” (AND is the Boolean operator; source: Web of Science, updated to 8 January 2025).

Despite the potential and reliability of DoE, this methodology has rarely been utilized in hybrid organic-inorganic systems. Indeed, sol-gel systems involve many operative conditions (e.g., temperature, pH, chemistry of the sol-gel precursors, sol-gel routes, sol-gel recipes, …), which limit an effective DoE exploitation. As a consequence, it is possible to use DoE for identifying the necessary experimental tests, though their number is still quite high and tests are still time-consuming. To try to fix this issue, during the last five years, a new approach based on the use of AI [24,25,26] through the exploitation of ML methods [27,28] has emerged (and is emerging). Although it is still in its infancy (Figure 1), this new methodology seems very promising and intriguing, specifically referring to materials science and technology. This new approach involves teaching AI how to exploit already existing experimental data (even incomplete) related to material systems similar to the one being explored to predict key parameters of this latter, minimizing the error while maximizing their reliability.

This review work aims to summarize the research efforts spent so far on the use of AI/ML methods in the field of sol-gel-derived hybrid materials, underlining the current achievements and providing the reader with some perspectives about the development and implementation of this new research approach. To this aim, first, the fundamentals of AI and ML tools will be described; then, the main research outcomes on the use of AI and ML tools for predicting some key properties of hybrid organic-inorganic sol-gel-derived systems will be discussed.

## 2. Artificial Intelligence and Machine Learning in Materials Science: An Overview

The next paragraphs will summarize the basic concepts of AI and ML tools for those who are not familiar with them, demonstrating that, from an overall point of view, this new strategy can be successfully applied to materials science.

ML is changing the world and science, opening new avenues for the development of innovative and functional materials. Starting from atomistic behavior and physicochemical data, it is possible to predict selected material properties using specific models [29]. Such models can be complex algorithms employed in imaging or material characterization, which can provide data-driven insights in the analysis of material properties and shed light on trends and not-obvious correlations [30]. Predictive models are fostering material discovery and optimization, also enabling the preparation of new compounds with customized features in diverse fields [31]. While, on one hand, the application of wide datasets and ML is making it possible for computers to learn as they progress, on the other hand, it is removing the need for long procedures based on trial and error, expensive routes, and dangerous synthesis steps along the fabrication of products. ML algorithms can recognize patterns, help in risk assessment, adapt over time, and extrapolate useful insights for the design of experiments [32]. ML is a subfield of computer science dealing with AI, and it mainly searches to reveal patterns from data to enhance performance in different activities.

### 2.1. Supervised Learning Approaches

**Figure 2:**
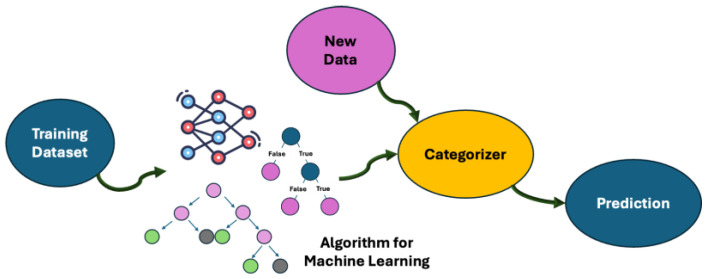
Supervision approaches for the learning process.

During the design of a new material, many limitations or constraints can be found; thus, ML is often employed to overcome these adverse factors. In this context, supervised learning is identified as the most representative form of ML [33]. This learning approach involves two steps: the algorithm reveals patterns in the training dataset, which may consist of labels or sample sets, and then it converts patterns into a mathematical function, namely the model, during the training stage. Supervised learning models are generally implemented for predictions about samples that the models have not encountered in the inference stage [34]. To give an example, a typical learning task, following a supervised approach, is the classification problem, in which the learner approximates the behavior related to a function [35]. Training and test stages represent the crucial points of an ML process. In the training phase, the algorithm is built by the exploitation of examples gathered from the training dataset as input, so the acquisition of features from the learner or learning model can take place. Once labeled data are available for training, supervised learning can be applied, for example, in regression (e.g., predicting the prices of products on the market) and classification (e.g., image classification) tasks [36]. The training of linear regression, decision trees (DTs), support vector machines (SVMs), logistic regression, random forests (RFs), and neural network (supervised ones) algorithms is performed on labeled data, thus learning the mapping between the input parameters and their related outputs (see Figure 2) [33].

In artificial neural networks (ANNs), a hidden layer is a layer composed of artificial neurons, which is different from both the input layer and the output layer, as it appears clear in multilayer perceptrons. Hidden layers transform inputs into a prediction or outputs through weighted connections and activation functions. Hyperparameters, which control the learning process of an ML model, are set before the model starts its training and are different from the internal values learned during the training. The optimization of the hyperparameters allows for the maximization of the model’s performance. In a typical ML process, the biases and weights regulate the input of data flow (forward propagation) through the layers of a neural network. Biases and weights are started and then iteratively updated during the training stage by backpropagation [37]. During the backpropagation, the prediction is compared to the actual target value to obtain an error, which is then minimized by the network by adjusting weights and biases. This way, the model learns and improves its prediction accuracy.

Support vector machine (SVM) is a learning model that can be adopted to address and solve regression and classification problems by supervised learning. This model is especially applied to minimize structural risk and find the best hyperplane dividing a dataset into different classes. Its parameters (e.g., kernel and penalty) influence the effectiveness of prediction models. SVMs can employ a kernel trick to convert data into higher dimensions to optimize the splitting of data when these latter are not linearly separable [38]. For this reason, SVMs are more effective and accurate when: (i) the classes are very well separated, (ii) they are implemented for classification tasks in high-dimensional spaces (e.g., text categorization, bioinformatics), and (iii) they are employed to estimate implicit functions [39,40].

### 2.2. Unsupervised Learning Methods

In materials science, the possibility to look through extensive quantities of data by using ML tools is becoming increasingly important. Unsupervised learning searches for features without manual analysis or explicit supervision. Such a type of learning is useful to find structure or hidden patterns within unlabeled data [41]. To handle customer segmentation or data compression, algorithms based on unsupervised learning (e.g., K-means, principal component analysis (PCA), and generative adversarial networks) are valuable tools [42].

### 2.3. Deep Learning Methodologies and Bayesian Methods for Optimization

Deep learning is composed of interconnected classifiers based on activation functions and linear regression [43]. Among the existing ML approaches, shallow learning and deep learning differ in their ability to process complex data. More specifically, shallow learning applies models made of one or two hidden layers (i.e., nonlinear feature conversion layers) to handle tasks with simple relationships in data. On the other side, deep learning employs more complex models with multiple hidden layers to learn intricate patterns in data. The most used shallow models are Gaussian mixture models, support vector machines, and logistic regression [44]. For pattern recognition tasks (e.g., image recognition, speech recognition), deep learning is the most appropriate, as it allows for easy management of huge amounts of data. The most frequently employed deep learning algorithms are recurrent neural networks for sequential data, conventional neural networks for image-related tasks, and generative pre-trained transformer models for natural language processing [45]. Bayesian optimization methodology allows for setting parameters for the optimization of the decision-making process by implementing an objective function. Bayesian methods (e.g., Bayesian linear regression and Bayesian networks) are mostly used to model uncertainty based on evidence and objective functions by employing probability [46]; therefore, they are suitable for tasks dealing with decision-making under probabilistic reasoning or in cases where assumptions necessitate being included into the model, for example, in natural language modeling [47,48]. Bayesian optimization strategy optimizes black-box functions (i.e., functions where the internal code and inner workings are hidden; this means that the only interaction of the user with the function concerns the feeding of input and the observation of the outputs), and it represents a valuable tool to find optimal configurations for ML models, especially when the analytical form of the functions is unknown, derivatives are not reliable, and evaluations are expensive. As an example, Gaussian process regression (GPR) is a Bayesian and non-parametric approach to regression, which is mostly appropriate to predict an estimation of the uncertainty, obtain a flexible data-driven model, and model functions with unavailable derivatives.

### 2.4. Artificial Intelligence in Materials Science and Property Prediction

**Figure 3:**
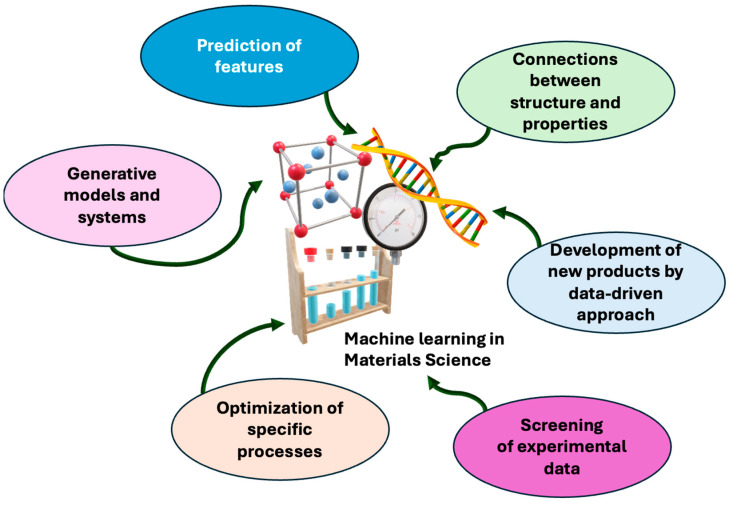
Main routes for exploiting artificial intelligence in materials science.

ML is mostly employed in materials science to predict a material’s properties or design new routes of preparation. The algorithm for the prediction is generally built based on an extensive amount of material data. The input dataset is used to train the algorithm, which can then forecast the features of new and untested materials (or intermediates), also along with their synthesis. The proper algorithm can correlate patterns behind the data collected during the experimental work or available in the literature. The possibility to define the future properties of novel materials during their design or manufacturing allows for the identification of the most profitable routes of preparation, screening steps with fewer endeavors, and cost-saving decisions. Thus, the task of discovering the optimal compositions to obtain materials with desired properties can be faced by the ML approach. ML algorithms can be optimal to search for non-linear connections involving several variables concerning the synthesis and characterization of polymers, 2D materials, organic-inorganic structures, and multi-component compounds [49,50]. A huge number of properties have been predicted by ML using wide input datasets (e.g., made of different physico-chemical properties), such as defect energetics [51], glass transition temperatures [52], mechanical properties [53], thermal conductivity [54], and catalytic action [55]. Figure 3 displays how AI finds the way to be applied in material synthesis.

### 2.5. Other Machine Learning Algorithms and Statistical Tools for Data Optimization

Adaptive boosting is a well-known ML method that is commonly employed for classification tasks, but it can also be useful for regression [56]. This method forms strong classifiers through the combination of multiple weak learners. Boosted regression trees, or gradient boost (GB) regression trees, are used to connect decision trees and promote the performance of regressions and classifications [57]. Similarly to adaptive boosting, GB regression trees are built by weak learners (e.g., small regression trees) to forecast a continuous target variable: practically, each new tree tries to correct the errors of the combined existing trees. K-nearest neighbors (K-NN) is a non-parametric algorithm applied for both regression and classification, which makes predictions based on the closeness of data points [58]. K-NN starts from the assumption that similar data points are located close to each other in feature space; therefore, such an algorithm stores the whole training dataset and performs the predictions considering the majority class or the average value of the K closest training examples to the input point. Long short-term memory (LSTM) algorithms are used to counter the issue related to the vanishing gradients in deep learning [59]. They are recurrent neural network architectures able to handle prediction tasks like time series forecasting and natural language processing. LSTM finds a wide application with sequential data and in tasks where previous time steps affect future predictions. Finally, among dimensionality reduction (i.e., reduction in input features in datasets, preserving as much variation as possible) techniques, the principal component analysis is frequently implemented in data science to transform large datasets into a set of orthogonal axes (i.e., principal components), which extract the most important variance within the data [60]. This results in a better visualization of high-dimensional data and in an improvement of ML models’ performance.

### 2.6. Statistical Metrics and Performance Parameters for Prediction Evaluation and Data Correlation

Several statistical metrics are generally employed to quantify the performance efficiency of prediction models [61,62]. These metrics give a clear idea of the accuracy and errors of the models in different tasks (e.g., classification, regression). The application of regression models allows for predicting continuous values. Mean absolute error (*MAE*) can be used to measure how close the actual values are to the predicted ones, as it provides the absolute differences between both values:

(1) $$MAE=\frac{1}{n}\sum_{i=1}^{n}\left|y_{i}-{\hat{y}}_{i}\right|$$

where $y_{i}$ is the actual value, ${\hat{y}}_{i}$ is the predicted value, and *n* is the number of data points. Good prediction accuracy corresponds to small *MAE* values. Mean squared error (*MSE*) can be a valuable alternative to *MAE*:

(2) $$MSE=\frac{1}{n}\sum_{i=1}^{n}{\left(y_{i}-{\hat{y}}_{i}\right)}^{2}$$

However, *MSE* emphasizes larger errors more than smaller ones because of the squaring, and it is more sensitive than *MAE* to outliers. Root mean squared error (*RMSE*) represents the square root of *MSE*:

(3) $$RMSE=\sqrt{\frac{1}{n}\sum_{i=1}^{n}{\left(y_{i}-{\hat{y}}_{i}\right)}^{2}}$$

This statistical metric transforms the units to match those of the original data. Also, lower values of *RMSE* indicate better accuracy. *R*-Squared (*R*^2^) indicates the proportion of the variance included in the dependent variable that is possible to predict from the independent variable:

(4) $$R^{2}=1-\frac{\sum {\left(y_{i}-{\hat{y}}_{i}\right)}^{2}}{\sum {\left(y_{i}-\bar{y}\right)}^{2}}$$

where $\bar{y}$ is the mean of actual values. *R*^2^ can assume values ranging from 0 to 1, with 1 referring to a very reliable prediction. Particularly, a high *R*^2^ reveals that the model well explains most of the variance in the target. Mean absolute percentage error (*MAPE*) can be very useful; it immediately gives an idea about the prediction accuracy of the model as a percentage:

(5) $$MAPE=\frac{100}{n}\sum_{i=1}^{n}\left|\frac{y_{i}-{\hat{y}}_{i}}{y_{i}}\right|$$

The adoption of such a statistical metric is suggested when the actual values are very small. Due to their definitions, *MSE*, *MAE*, and *R*^2^ are mostly suitable for regression tasks. Concerning classification tasks, the final objective is to predict discrete labels, and thus the accuracy factor is generally employed to evaluate the prediction performance of the algorithm:

(6) $$Accuracy=\frac{True\;Positives+True\;Negatives}{Total\;Instances}$$

High accuracy means that most of the predictions performed by a specific model are correct [63]. Kling-Gupta efficiency (KGE) is a metric applied to study the performance of continuous time series models through the comparison of simulated values to observed ones [64]. KGE enables the contributions of different components of model performance to be separated, allowing the reasons for good or poor performance to be understood. *R*-value, also called the correlation coefficient, measures the strength of the linear relationship linking two variables. This statistical metric is used in both correlation analysis and regression to estimate the degree of relation between two variables. The strength of the linear relationship between the variables increases with the *R*-value’s proximity to either +1 or −1 [65].

## 3. Current State-of-the-Art in the Use of AI/ML Approaches for Predicting Key Properties of Hybrid Sol-Gel-Derived Materials

The use of ML in the development of new materials is expanding. Sol-gel chemistry enables the preparation of materials, especially glasses and ceramics, by transforming a “sol” into a “gel” through hydrolysis and condensation reactions. This method allows for the synthesis of various systems, including nanoparticles, thin films, and monolithic objects, often at room temperature. Such types of materials are among the most commonly explored and fabricated sol-gel products. In addition to the prediction of materials’ properties and advantages under operative conditions, ML models can contribute to emphasizing the valuable characteristics of the sol-gel method, namely: mild synthesis conditions, versatile chemistry, and high sustainability.

### 3.1. Sol-Gel-Derived Aerogels: Machine Learning Prediction of Their Physico-Chemical Features

Silica aerogels are porous sol-gel systems exhibiting unique characteristics, such as ultra-low density, high surface area, low thermal conductivity, and high porosity. To describe silica aerogels at the nanoscale and their related fractal structure, fractal geometry is generally employed. Abdusalamov et al. proposed an ANN to predict the fractal dimension of silica aerogels and reconstruct their microstructures from a given fractal dimension [66]. The diffusion-limited cluster–cluster aggregation (DLCA) approach was applied to obtain the microstructures [67]. In the forward propagation model, DLCA network structures were used as input parameters to train the ANN. The latter was able to predict the fractal dimension for any given set of input parameters with an accuracy of *R*^2^ = 0.973. On the other side, in back-propagation mode and by including model constraints in the inversion algorithm, the same ANN was inverted and employed to predict input DLCA model parameters for a desired fractal dimension with an error of 2% [66].

ANNs are not the only ML tools able to support the development and characterization of such materials. As some of the major applications of silica aerogels are as electrodes in the field of energy conversion and storage and high-end adsorbers, Scherdel et al. proposed the implementation of linear regression and GPR ML models to predict the solid-phase thermal conductivity of silica aerogels from a structural dataset [68]. The research group built the structural input dataset from small-angle X-ray scattering data and physical properties of the aerogels, also using different data subsets as predictors, based on different states of synthesis (i.e., wet and dry), to evaluate the model performance [68]. To give an idea of model evaluation, after supercritical drying, the linear regression model gave an RMSE of ~2 for the training and ~10 for the prediction of the solid-phase thermal conductivity. Considering that the morphology of gels changes a lot during the processing (e.g., supercritical drying), GPR models combined with SAXS data led to the best prediction.

As the properties of silica aerogels are strongly related to the synthesis pathway, it would be beneficial to predict the final features of such materials based on experimental data and operative conditions. In this context, Walker et al. developed a silica aerogel graph database (regarding the synthesis conditions of 10^3^ silica aerogels) and a supervised ML neural network regression model to better understand and visually display the relationship between synthesis conditions and final properties of aerogels [69]. By following a validation test, the model maps could predict the aerogel surface area from processing and optimal synthesis conditions with an error lower than 5%, while the graph database was able to reduce time and experimental dimensionality during the preparation of high surface area silica aerogels for application in thermal insulation or sorption media. The silica aerogel graph database was used as an input dataset after removing statistical outliers and aerogels with high prediction errors [69]. This data cleaning process could enhance the model, leading to the prediction of aerogel surface area with an average error of 109 ± 84 m^2^/g (112% ± 72.6%). The normalized predicted versus actual graph for the surface area values of aerogels in the model, using K-fold cross-validation (i.e., a resampling and sample splitting method that uses different portions of the input data to test and train the model on different iterations), gave *R*^2^ of 0.731, *MSE* value of 0.014, and *RMSE* of 0.118 [69].

Despite the excellent properties of silica aerogels, their mechanical brittleness results in significant challenges. It is well known that surface area, pore volume, density, and thermal conductivity strongly influence the final properties of silica aerogels [70]. However, the silica content may also remarkably affect the performance of such aerogels. Chaouk et al. explored the feasibility of PCA to investigate the influence of silica content on the physicochemical properties of aerogels [71]. Also, cross-correlation analysis was employed to find the relationships between surface area, pore volume, density, and thermal conductivity. PCA results confirmed that silica content is the prominent parameter impacting the aerogel properties, in agreement with the first principal component giving a positive correlation (*R*^2^ = 94%) with the silica content. By avoiding long screening experimental procedures, the research group could demonstrate that higher silica contents correspond to lower thermal conductivity, surface area, and porosity [71]. Finally, the outcomes of the second principal component suggested the thermal conductivity as crucial in influencing the final properties of aerogels, especially in samples with moderate/high silica loadings. Chaouk et al. applied PCA to reduce the dimensionality of the dataset and effectively understand the complex interaction between surface area, porosity, density, thermal conductivity, and silica content of aerogels. PCA results were found optimal, as the statistical method could capture 97.45% of the total variance [71].

### 3.2. Sol-Gel Derived Hybrid Nanofluids: Machine Learning Prediction of Their Physico-Chemical Properties

Conventional heat transfer liquids (e.g., water, ethylene glycol) show lower thermal conductivity than solid particles. To enhance the thermophysical properties of traditional fluids, the scientific community has proposed different solutions, for example, turbulators and nanofluids. Nanofluids can be prepared by suspending metallic, ceramic, and non-metallic nanoparticles in a base fluid and can guarantee high heat transfer performance [72,73]. Researchers are progressively looking for new, effective, stable, and well-designed nanofluids suitable for fluids with specific density and viscosity. In this context, Said et al. produced, starting from graphene oxide (GO), rGO-Fe_3_O_4_-TiO_2_ ternary hybrid nanocomposites by sol-gel methodology to obtain ethylene glycol-based stable nanofluids, whose viscosity and density were deeply investigated through the variation in temperature and volume concentration [74]. The use of analytical methods for the optimization of viscosity and density of the rGO-Fe_3_O_4_-TiO_2_ hybrid nanofluids may involve long operations to handle a large volume of nonlinear experimental data. To avoid such a time-consuming and expensive procedure, Said et al. employed advanced ML techniques like boosted regression tree (BRT), SVM, and ANNs to predict the influence of temperature and volume concentration on the viscosity and density of rGO-Fe_3_O_4_-TiO_2_ hybrid nanofluids. To have an idea about the prediction performances, statistical errors were calculated to compare predicted values with experimental outcomes [74]. The *R*-values of BRT-based density (0.9989) and viscosity (0.9979) were higher than those of the ANN-based and SVM-based prediction models. These results, together with the values of *R*^2^ (BRT > ANN > SVM) and KGE (BRT > ANN > SVM), demonstrated the higher efficiency of BRT-based prognostic models. Also, the BRT-based models reported lower *MAPE* and *RMSE* than both ANN and SV algorithms. Finally, the research group highlighted that BRT, ANN, and SVM could accurately predict the experimental values of density and viscosity of ternary hybrid nanofluids along wide ranges of temperature and nanoparticle concentration ratio [74].

Among its possible uses, GO can be adopted for the fabrication of water base hybrid nanofluids by combining it with suitable metal oxides. Lately, Kanti et al. prepared hybrid nanofluids by mixing CuO and GO at different ratios. CuO and GO were synthesized by sol-gel chemistry, and the effects of pH on thermal conductivity, stability, and viscosity of hybrid nanofluids were studied for each formulation and composition in a specific range of temperature [75]. The experimental results revealed that these hybrid nanofluids may be promising in solar power facilities, where ethylene glycol or molten salts often require the addition of proper nanoparticles to improve their thermophysical features. Bayesian optimized support vector machine (BoA-SVM) and Bayesian optimized boosted regression trees (BoA-BRT) were applied as ML models for the prediction of thermal conductivity and viscosity of hybrid nanofluids. The prognostic capabilities of models were evaluated by several statistical indices (e.g., *MSE*) [75]. The correlation in terms of R^2^ between observed and predicted values of thermal conductivity and viscosity was in the range of 0.9923–0.9922, while the prediction error, measured by MSE, was quite low (between 0.0000085 and 0.00354). BoA-BRT and BoA-SVM were proved as robust forecasting techniques for the prediction, with BoA-SVM (*R*^2^ = 0.9992) showing slightly superior efficiency than BoA-BRT (*R*^2^ = 0.9976). The research group demonstrated that ML can be useful to give a forecast about potential thermophysical parameters of hybrid nanofluids under a wide range of operative conditions, also reducing the implementation of expensive technologies, which are usually employed to test heat transfer fluids [75].

As additional proof that the combined use of sol-gel chemistry and ML can be the needle of the scale in the development of new hybrid nanofluids, Kanti et al. proposed water-based hybrid nanofluids composed of Al_2_O_3_ and GO at different mixing ratios, also studying their thermophysical characteristics and dispersion stability. Al_2_O_3_ and GO nanoparticles were synthesized by sol-gel routes and Hummer’s method [76]. The stability of the hybrid nanofluids was tested by the incorporation of three different surfactants, while the effects on both thermal conductivity and viscosity were measured at different temperatures (30–60 °C) and filler concentrations (0.1 to 1 vol.%) [76]. The research group found that mainly the inclusion of GO content could increase the thermal conductivity and the viscosity of hybrid nanofluids. Finally, regression models (i.e., a Bayesian optimized support vector machine (BoA-SVM) and a Bayesian optimized wide neural network (BoWNN)) were developed to predict the thermal conductivity and the viscosity of hybrid nanofluids. The prediction of the thermophysical properties achieved a good prognostic efficiency of 97.15–99.91% [76].

### 3.3. Machine Learning Assisted Sol-Gel Methodologies for the Preparation and Characterization of Functional Polymeric Products

Among the possible materials made by sol-gel chemistry, functional nanostructures composed of different inorganic phases can be employed to fabricate low-energy surface coatings [77]. These coatings are mostly used to generate hydrophobic and superhydrophobic surfaces on a single substrate. In this context, ML tools can predict the optimal set of parameters to obtain hydrophobic surfaces for a given substrate. Manoharan et al. developed an ANN to predict the different factors affecting the anti-wetting behavior and surface contact angle of coated printable paper substrates [78]. The research group used the outcomes of this prediction to optimize the coating method (i.e., dip coating, spray coating, spin coating, and inkjet printing), the number of deposited layers, the selection of proper silane (i.e., trimethoxymethylsilane, TEOS), and the sol-gel-derived material applied during the deposition stage. Manoharan et al. exploited base material, initial contact angle, and final contact angle on the front side and back side as input data for the ANN, which was trained for up to 5 cycles until the convergence (*R* ≈ 1) of all parameters. The ANN implemented a tangent sigmoid as a transfer function and the Levenberg–Marquardt algorithm (i.e., an algorithm used to solve non-linear least squares problems), involving Gauss Newton and Gradient methods, as the optimization technique. The contact angle resulting from the predicted parameters was found to be similar to the actual values, with a maximum error of ±4.5° [78].

**Figure 4:**
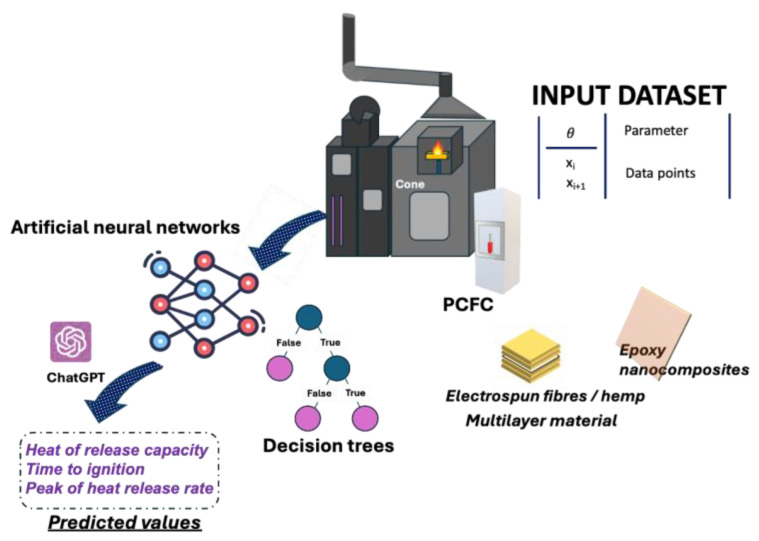
Development of hybrid epoxy nanocomposites and multifunctional textiles and prediction of their fire properties.

To be suitable for several industrial applications, polymeric composites must pass specific requirements in terms of fire behavior. As most polymers are highly flammable, the exploitation of flame retardants is generally needed [79]. As an example, the incorporation of magnesium hydroxide nanoparticles into the polymer matrix allows for a reduction in the heat release rate and smoke emission of final composites, also lowering their flammability. To test the fire performance of polymeric materials, a long series of disruptive and time-consuming fire tests must be performed to find the optimal formulation for a specific application [80]. ML tools can help to expedite the development of new flame-retardant polymeric composites or support their characterization by predicting key fire parameters. Recently, Bifulco et al. synthesized an in-situ hybrid Mg(OH)_2_-epoxy nanocomposite through sol-gel methodology [81], also predicting its heat release capacity through the implementation of a made-on-purpose fully connected feed-forward ANN (see Figure 4) [82]. From the experimental side, nanocomposites incorporating ~5 wt.% of in-situ generated Mg(OH)_2_ nanoparticles exhibited a significant decrease in the HRC (around −34%), measured by performing a certain number of pyrolysis combustion flow calorimetry tests, and in some cone calorimetry parameters (e.g., peak of heat release rate (pkHRR), heat release rate, and total smoke release) [82]. The developed ANN-based system could predict the HRC of the prepared Mg(OH)_2_-epoxy nanocomposite, giving very low error values (i.e., MAE and RMSE equal to 145.6 and 186.1, respectively).

Sol-gel chemistry has also been combined with a DCNN (deep convolutional neural network) algorithm: this latter applies a convolution operation over one-dimensional sequence data, which are commonly adopted to analyze temporal signals or text. A one-dimensional convolutional neural network (1DCNN) in deep learning is specifically designed for processing one-dimensional sequence data [83]. To this aim, Ma et al. developed a system for the shelf-life estimation of food products, namely the freshness-related information of salmon for consumers [84]. The system was fabricated by incorporating a dye-containing, optically changeable colorimetric sensor into a sol-gel functionalized paper sensor using a DCNN model based on images for the freshness estimation. The cross-reactive paper sensor array was obtained by employing different dyes and treating paper with sol-gel particles (synthesized from (3-aminopropyl) triethoxysilane -APTMS- and TEOS) and indicators (e.g., ammonia gas indicator, pH indicator, indicators for volatile organic compounds, and oxidation indicator) [84]. The augmentation by ML allowed for setting the limit for ammonia detection at ~17.1 ppm and the accuracy up to 99.2% for the non-destructive estimation of the salmon freshness (i.e., visual and olfactory), in agreement with the requirements of food supply chains and scalable batch production [84].

Another interesting application of AI/ML tools refers to electrospinning and sol-gel-derived nanostructures, which can be used to develop polymeric nanofibers with several functional properties. In this context, Bifulco et al. prepared a multilayer material made of coated hemp blankets with cross-linked electrospun polyvinylpyrrolidone-silica blankets and TiO_2_ nanoparticles (Figure 4) [85]. This MM showed good tensile properties and could work not only as a smoke suppressant but also as a fireproof fabric. The assessment of the fire properties is usually time-consuming and requires the preparation of a huge number of precious samples. Despite the incomplete input dataset consisting of fire parameters related to sol-gel-treated textiles, and thanks to the support of ChatGPT (GPT-4o version), the research group implemented ANNs and decision trees to predict the time to ignition (TTI) and the pkHRR of the MM. The accuracy of the prediction was evaluated by the calculation of MAPE values, considering the experimental data and the predicted values. Decision trees gave *MAPE* values of 0.431 (~43%) and 0.336 (~34%) for TTI and pkHRR, respectively. Conversely, *MAPE* values assessed by the outputs of ANNs were 0.468 (~47%) for the TTI parameter and 0.404 (~40%) for the pkHRR parameter [85]. Based on these results, for both algorithms, a reasonable predictive capability was found [86]. However, decision trees provided the lowest *MAPE* values, hence becoming preferable and more reliable in case of some missing input data.

Hybrid sol-gel materials combine the best features of the organic and inorganic phases in a single material due to the peculiarities of the resulting co-continuous network. In this respect, Lin et al. demonstrated that sol-gel chemistry and ML can be applied to obtain large hybrid monoliths, made of silica/poly (tetrahydrofuran), with good mechanical properties as well as resistance toward cracking phenomena [87]. These latter are commonly triggered by the combined action of capillary and shrinkage stresses inside the monoliths, which mostly take place during the drying procedure. The shrinkage along different drying processes was assessed by collecting computational scribe calculations on X-ray micro-computed tomography images. A filtered back-projection algorithm was applied to reconstruct captured projections into a 3D image matrix. A random forest voxel classifier (i.e., an ML algorithm) was implemented to segment hybrid samples within the 3D images [87,88].

### 3.4. Sol-Gel Derived Metal Oxides, Organic Structures, and Inorganic Materials for Several ML-Optimized Technologies

**Figure 5:**
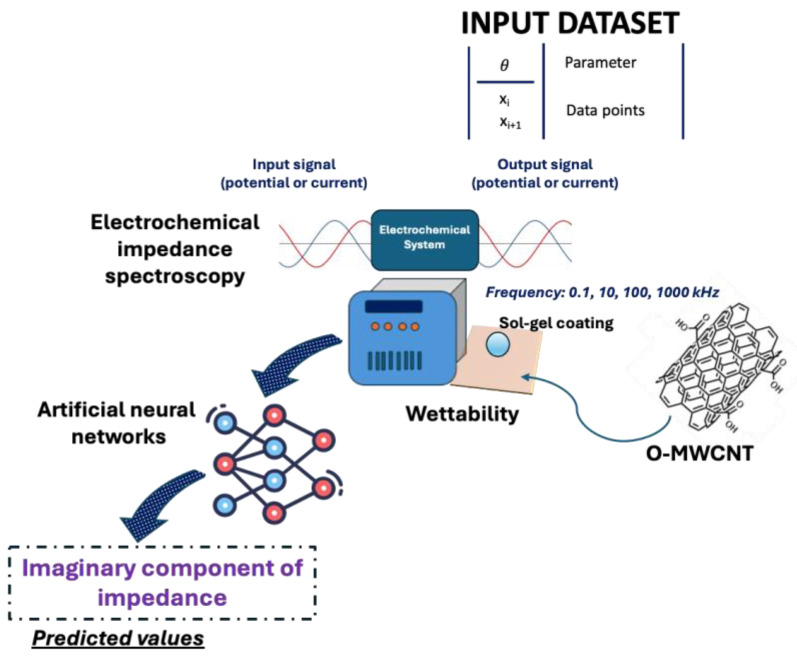
Development of sol-gel coatings containing O-MWCNTs and prediction of the imaginary part of the impedance for corrosion resistance technologies.

Many sol-gel techniques can be employed for the manufacturing of coatings able to confer very low wettability and chemical resistance to different substrates [89]. To give an example, in the oil and gas sector, the wettability of surfaces in pipelines affects the corrosion rates; therefore, several strategies are usually adopted to limit the occurrence of material deterioration phenomena [90]. Akbarzadeh et al. obtained sol-gel coatings starting from TEOS, methyltriethoxysilane, and glycidoxypropyltrimethoxysilane as precursors (Figure 5) [91]. The research group incorporated different amounts of oxidized multi-walled carbon nanotubes (O-MWCNTs) to modify the corrosion resistance properties and hydrophobicity, and increase the barrier effect of the silane-based nanocomposite coatings, also checking the performance through electrochemical impedance spectroscopy and wettability measurements. By using a single hidden-layer perceptron and the Levenberg–Marquardt algorithm optimization procedure as a learning algorithm, an ANN model was constructed to predict the imaginary part of the impedance [91]. The immersion time of mild steel panels in the sol-gel silane solution by dip-coating, the different loadings of O-MWCNT in the solution, and the real part of the impedance were implemented as input data. The accuracy of the optimized model was calculated by comparing the experimental data with the predicted parameters. High O-MWCNT loadings in the silane-based coatings resulted in a decrease in the corrosion current density, as confirmed by polarization curves and AC impedance spectra. The ANN model demonstrated highly reliable predictive efficiency, as the statistical errors, including *MSE* and *MAE*, were 0.00099606 and 0.0106, respectively. Also, the R^2^ value was found to be around 0.9879, revealing the absence of scattering in the entire data prediction range [91].

Further, AI/ML tools can support sol-gel chemistry along with the synthesis of specific nanostructures that are crucial for the preparation of biosensors [92]. Biosensors’ receptors include enzymes, nucleic acids, and microorganisms, among others. To open new perspectives in the fabrication of nanomaterial-based sensors, Zalke et al. proposed an ML-assisted screen-printed and non-enzymatic biosensor for the quantification of urea concentrations, which is very important during medical intervention [93]. For the detection of urea, the biosensor was tailored by an MWCNT-ZnO nanocomposite functionalized with CuO micro-flowers (MFs). CuO-MFs were prepared by the sol-gel approach starting from copper nitrate trihydrate as a precursor. To have an idea about the sensor’s performance for the detection of urea, cyclic voltammetry at different scan rates was applied to assess the dependence of peak currents on analyte concentration. The non-enzymatic biosensor exhibited good sensitivity and a satisfactory linear range of operation [93]. Regression-based ML models were employed to predict CV parameters for the optimization of the measured experimental concentrations of urea. To improve the sensor’s accuracy and determination of the urea concentration, linear regression, DT, K-nearest neighbor (KNN), RF, adaptive boosting (AdBoost), and GB models were used, and their outcomes were compared by different regression metrics. ML models outperformed linear regression in predictive accuracy metrics, e.g., *RMSE* and *R*^2^. For example, the KNN model gave a significantly lower *RMSE* (0.0004) than linear regression (0.0006), together with a higher (0.981) *R*^2^ compared to linear regression (0.953) [93]. Thus, the research group demonstrated that the KNN model could well predict optimal CV parameters and thus the sensor’s response to different urea concentrations, not only providing a better fit of the data but also capturing more variance.

Water is a precious resource for humans, and its proper management is getting increasingly complicated, as new pollutants and persistent contaminants are constantly found during analysis [94]. In the field of water decontamination, green strategies or waste-to-wealth approaches allow for the synthesis of functional nanoparticles by the sol-gel method [95]. ZnO nanoparticles can be very effective in addressing groundwater contamination and countering microbial cells. Chelghoum et al. obtained ZnO-based nanoparticles by using a sol-gel route and lemon peel aqueous extract [96]. ZnO-based nanoparticles were employed to photodegrade quinoline yellow dye by sunlight irradiation while studying their antioxidant and antibacterial properties. The nanoparticles exhibited a notable photoactivity, also revealing high efficiency as antioxidants and against Gram-positive bacterial strains. The photodegradation action was mainly affected by the initial concentration of the quinoline yellow, the pH, and the catalyst dosage. To predict the photodegradation rates of quinoline yellow, the research group developed a GPR combined with an optimization Lévy flight distribution algorithm [96]. The implementation of an ARD-Exponential kernel function (i.e., a tool enabling the non-linear transformation of data without explicitly performing such transformations, also mapping input data into a high-dimensional feature space) allowed us to achieve high accuracy. The prediction model gave high correlation coefficients (i.e., close to 1) between predicted values and the actual ones and low *RMSE* values along all phases (i.e., 0.0160, 0.0250, 0.0244, and 0.0179 during training, testing, validation, and for the whole dataset, respectively), confirming good reliability in forecasting photodegradation rates of quinoline yellow by the synthesized nanoparticles. The prediction of dye photodegradation could avoid time-consuming procedures and waste of resources [96].

Furthermore, nanostructured organic-inorganic hybrids made by ML-assisted sol-gel procedures can represent potential solutions and facilitate new advancements for water-splitting studies [97]. In this context, Koyale et al. improved the photoelectrochemical activity of graphitic carbon nitride (g-C_3_N_4_) nanosheets through the synthesis of their nanocomposites with metal-organic framework-derived porous CeO_2_ nanobars along with ZnO nanorods and TiO_2_ nanoparticles [98]. Such synthesis could enhance the photoelectrochemical behavior of g-C_3_N_4_ by lowering the charge recombination rate. CeO_2_ nanobars and g-C_3_N_4_ nanosheets were prepared starting from their precursors, whereas the sol-gel method was applied to obtain ZnO nanorods and TiO_2_ nanoparticles. The binder-free brush-coating technique was deployed to manufacture nanocomposites-based photoanodes, which were analyzed through several electrochemical experiments [98]. The binary g-C_3_N_4_/CeO_2_ nanocomposites, including 20 wt.% CeO_2_ nanobars (gC20 nanocomposites), showed a significant enhancement in the current density compared to that of bare g-C_3_N_4_ nanosheets. A further improvement in the current density was detected using ternary gC20/TiO_2_ (gCT50) and gC20/ZnO (gCZ50) nanocomposites: this finding was mainly ascribed to a lower charge transfer resistance and an increased donor density. The research group also predicted the stability (i.e., the values of current density over time) of gCZ50 and gCT50 nanocomposite-based photoanodes by the implementation of a recurrent neural network-based LSTM technique [98]. This technique could effectively forecast the stability of photoanodes, providing useful details about their durability. The performance efficiency of the prediction was evaluated by the calculation of *RMSE* and *MSE* metrics: both showed very low values for the training datasets (80% of the experimental electrochemical observations) and the stability prediction results.

Then, ML, with its data-driven approach, has successfully been exploited in the design of materials for batteries. Owing to such interest, Seitz et al. developed an approach using data obtained from small-angle X-ray scattering measurements for the manufacturing of optimized sol–gel Si-based porous materials for battery anodes [99]. The research group employed a feed-forward ANN connected to a pretrained autoencoder to map parameters (e.g., network topologies) related to the material synthesis to the SAXS curve of the resulting product. The trained network could give information about which parameters allow for a desired SAXS curve [99]. It was found that the prediction error of the ANN model could be reduced by widening the input dataset (during the design of the experiment stage) and training each output variable with an independent ANN algorithm, hence following a multi-step method for the material development.

Recently, the 1DCNN algorithm was also applied to prepare a sensor array able to effectively classify mixed gases. Such a sensor may be promising for developing electronic nose systems with low data processing and high sensitivity. Mu et al. fabricated a ZnO/SnO_2_-based micro-electromechanical system gas sensor array by inkjet printing sensing materials onto a micro-hotplate [100]. By combining the use of a pattern recognition algorithm and having time series input lengths as starting data, the sensor could identify formaldehyde, toluene, ammonia, and their binary and ternary mixtures. A sol-gel route was employed to prepare the pure ZnO precursor ink. Unlike traditional ML models, the pattern recognition unit based on the 1DCNN algorithm could avoid the manual feature extraction step, prevent any alteration in the original features of the data, and reduce the experimental work [100]. Furthermore, unlike the best-performing traditional algorithm, i.e., the SVM model, which gave an 80% recognition accuracy of the gas mixtures, the 1DCNN could achieve 99.8%.

To make better use of the systems discussed above, Table 1 summarizes their main features and outcomes.

## 4. Conclusions and Perspectives

Undoubtedly, combining AI with ML tools offers big potentialities as a valuable, reliable, and efficient strategy for predicting several properties of material systems.

This review paper first set out how AI and ML can be successfully applied to materials science, focusing particularly on hybrid organic-inorganic systems derived from sol-gel processes. Although work in this area is still in its infancy, predicting the properties of these hybrid systems while minimizing errors and maximizing the results’ reliability is of extreme interest. This is also relevant in limiting the time and energy spent on experimental investigations. It is worth noting that using a combined AI and ML approach does not mean avoiding experimental testing activities but rather being able to exploit this new strategy to save time and maximize the optimization of research outcomes.

Comparing the potential of AI/ML tools with DoE, it is worth noticing that the two strategies are completely different and rely on a very distinctive approach. DoE is applied to the system under investigation only, trying to minimize the experimental testing necessary to get an acceptable description of the system itself and using a “learning by doing” approach carried out by the investigator. Further, DoE often exploits a linear correlation among the identified variables that are characteristics of the investigated systems.

Conversely, AI/ML tools show the advantage of (i) using also non-linear correlations among the selected variables, (ii) exploiting datasets of material systems (similar to the investigated one) through, e.g., a data augmentation approach or generative models, and (iii) predicting the final properties of a material system by training algorithms on a based input dataset. This is surely a different strategy based on a “learning by doing” approach performed by an AI algorithm properly selected and managed by the operator.

Despite its high potential, this approach still has some limitations that currently prevent its widespread and effective use. From a general point of view, the combined exploitation of AI and ML requires access to a large dataset of properties of similar materials: indeed, the accuracy of predictions about the properties of the material under study depends heavily on the availability of reliable materials data. However, the availability of large datasets is not always guaranteed, which limits the use of this new strategy to a certain extent, even for sol-gel-derived systems. Furthermore, despite the significant research activity that has taken place in recent years, there is currently widespread skepticism surrounding the use of AI in applied sciences, particularly materials science. This is certainly slowing down the development and implementation of AI and ML tools in materials science. Using this new approach also requires cross-cutting skills related not only to sol-gel process chemistry and hybrid organic-inorganic systems but also to neural networks and decision trees. This requires complementary figures to be involved in the research groups.

In conclusion, despite the above limitations, these new approaches will likely see strong further development in the coming years, paving the way for their increasingly “intelligent”, reliable, and extensive use.

## Figures and Tables

**Table 1:** Sol-gel system technologies and outcomes related to the prediction of some specific properties and functions.

Sol-Gel System	Machine Learning and Optimization Tools	Predicted Parameters and Characteristics	Statistical Indices for Efficiency	Ref.
Silica aerogels	ANN	Fractal dimension Reconstruct micro-structures	R^2^ = 0.973	[66]
Silica aerogels	Linear regression Gaussian process regression	Solid-phase thermal conductivity	RMSE = ~2 for the training RMSE = ~10 for the prediction	[68]
Silica aerogels	Supervised ML neural network regression K-fold cross-validation	Surface area	R^2^ = 0.731 MSE = 0.014 RMSE = 0.118	[69]
rGO-Fe_3_O_4_-TiO_2_ hybrid nanofluids	BRT SVM ANN	Influence of temperature and volume concentration on the viscosity and density	R-values of BRT-based density (0.9989) and viscosity (0.9979) higher than those of the ANN-based and SVM-based models	[74]
Hybrid nanofluids based on CuO and GO at different ratios	BoASVM BoA-BRT	Thermal conductivity Viscosity	BoASVM (R^2^ = 0.9992) BoA-BRT (R^2^ = 0.9976)	[75]
Hybrid nanofluids composed of alumina (Al_2_O_3_) and graphene oxide (GO) at different mixing ratios	BoASVM BoWNN	Thermal conductivity Viscosity	Prognostic efficiency of 97.15–99.91%	[76]
Mg(OH)_2_-epoxy nanocomposite	ANN	HRC	MAE and RMSE equal to 145.6 and 186.1, respectively	[82]
Sol-gel functionalized paper sensor	DCNN model based on images Data augmentation	Non-destructive estimation of the salmon freshness	Accuracy up to 99.2%	[84]
Coated hemp blankets with cross-linked electrospun polyvinylpyrrolidone-silica blankets and TiO_2_ nanoparticles	ANNs Decision trees ChatGPT	TTI pkHRR	MAPE values of 0.431 (~43%) and 0.336 (~34%), respectively for the TTI parameter and the pkHRR parameter MAPE values assessed by the outputs of ANNs were 0.468 (~47%) for the TTI parameter and 0.404 (~40%) for the pkHRR parameter	[85]
Sol-gel coatings containing O-MWCNT nanotubes	ANN Levenberg–Marquardt algorithm	Imaginary part of the impedance	MSE and MAE were 0.00099606 and 0.0106, respectively R^2^ value was found around 0.9879	[91]
Xanthine biosensor based on Co_3_O_4_ nanoparticles and MWCNTs (tailored by a MWCNT-ZnO nanocomposite functionalized with CuO-MFs)	Linear regression DT KNN RF AdBoost GB	Optimization of the measured experimental concentrations of urea CV parameters	KNN model gave lower RMSE (0.0004) than linear regression (0.0006), together with a higher (0.981) R^2^ compared to linear regression (0.953)	[93]
ZnO-based nanoparticles	GPR Lévy flight distribution	Photodegradation rates of quinoline yellow	RMSE values along all phases (i.e., training (0.0160), testing (0.0250), validation (0.0244), and the whole dataset (0.0179))	[96]
ZnO/SnO_2_-based micro-electromechanical system gas sensor array	1DCNN	Classification of mixed gases	The conventional SVM model gave an 80% recognition accuracy of the gas mixtures, while the 1DCNN provided 99.8%	[100]

## Data Availability

No new data were created or analyzed in this study. Data sharing is not applicable to this article.

## Abbreviations

The following abbreviations are used in this manuscript:

Adaptive boosting	AdBoost
Artificial Intelligence	AI
Artificial neural network	ANN
Bayesian optimized boosted regression trees	BoA-BRT
Bayesian optimized support vector machine	BoASVM
Bayesian optimized wide neural network	BoWNN
Boosted regression tree	BRT
Decision tree	DT
Deep convolutional neural network	DCNN
Diffusion-limited cluster–cluster aggregation	DLCA
Gaussian process regression	GPR
Gradient boost	GB
Graphene oxide	GO
Graphitic carbon nitride	g-C_3_N_4_
Heat release capacity	HRC
K-nearest neighbor	KNN
Long short-term memory	LSTM
Machine learning	ML
Mean absolute error	MAE
Mean absolute percentage error	MAPE
Mean square error	MSE
Multiwall carbon nanotubes	MWCNTs
One-dimensional convolutional neural network	1DCNN
Oxidized multi-walled carbon nanotubes	O-MWCNTs
Peak of heat release rate	pkHRR
Principal component analysis	PCA
Random forest	RF
Root main square error	RMSE
Support vector machine	SVM
Tetraethylorthosilicate	TEOS
Time to ignition	TTI
